# Facing the Impact of the COVID-19 Pandemic: How Can We Allocate Outpatient Doctor Resources More Effectively?

**DOI:** 10.3390/tropicalmed7080184

**Published:** 2022-08-15

**Authors:** Xiaojing Hu, Hongjun Fang, Ping Wang

**Affiliations:** Medical Affairs Department, Peking University First Hospital, Beijing 100034, China

**Keywords:** COVID-19, outpatient numbers, patient reservation rate, doctor resources allocation, management strategy

## Abstract

The COVID-19 pandemic caused significant damage to global healthcare systems. Previous studies regarding COVID-19’s impact on outpatient numbers focused only on a specific department, lacking research data for multiple departments in general hospitals. We assessed differences in COVID-19’s impact on outpatient numbers for different departments to help hospital managers allocate outpatient doctor resources more effectively during the pandemic. We compared the outpatient numbers of 24 departments in a general hospital in Beijing in 2019 and 2020. We also examined an indicator not mentioned in previous studies, monthly departmental patient reservation rates. The results show that, compared with 2019, 2020 outpatient numbers decreased overall by 33.36%. Ten departments’ outpatient numbers decreased >33.36%; however, outpatient numbers increased in two departments. In 2020, the overall patient reservation rate in 24 departments was 82.22% of the 2019 reservation rate; the rates in 14 departments were <82.22%. Moreover, patient reservation rates varied across different months. Our research shows that COVID-19’s impact on different departments also varied. Additionally, our research suggests that well-known departments will be less affected by COVID-19, as will departments related to tumor treatment, where there may also be an increase in patient numbers. Patient reservation rates are an indicator worthy of attention. We suggest that hospital managers classify departments according to changes in outpatient numbers and patient reservation rates and adopt accurate, dynamic, and humanized management strategies to allocate outpatient doctor resources.

## 1. Introduction

Since the first confirmed case in December 2019, the novel coronavirus disease 2019 (COVID-19) pandemic has rapidly spread worldwide. To date, there have been nearly 500 million confirmed cases, resulting in more than 6 million deaths [1]. As the new coronavirus (2019 novel coronavirus) causes pneumonia [2], it is primarily transmitted through droplets released in the air when an infected person coughs, sneezes, or speaks. The most common transmission is via close contact (within 6 feet/1.83 m) [3]. This rapid and simple transmission facilitated a global outbreak. The devastating power of COVID-19 poses significant challenges to healthcare systems, economies, and social functioning worldwide [4,5,6]. Although governments have devoted substantial time and medical and financial resources to fight the pandemic [7], the vast majority of hospitals still struggle with inadequate medical staffing. However, problems faced by hospital managers involve much more than dealing with numerous COVID-19 patients. Many studies have found that, due to COVID-19’s impact, it is more difficult for patients to access outpatient services [8], and hospital outpatient numbers have significantly declined [9,10,11,12]. In the US, outpatient visits decreased by approximately 60% from the start of the pandemic to early April 2020 [10]. In the UK, one week after the national lockdown policy was enacted, national emergency department visits decreased by 25% [11]. A variety of departments, including orthopedics [13], dermatology [14], ophthalmology [15], rheumatic [16], pediatrics [17], stomatology [18], neurosurgery [19], otorhinolaryngology [20], mental health care [21], and contraceptive services [22] experienced declining outpatient numbers. Previous studies found that most countries adopted mandatory lockdown policies at different stages to avoid the spread of COVID-19 [23,24]; some hospitals delayed elective surgeries and non-emergency outpatient reservations [25,26] and started providing basic medical support to the public through telemedicine services [27]. In addition, many people feared becoming infected with COVID-19 in hospital [28] and preferred to self-medicate and monitor their health at home instead [29]. Rising unemployment rates and increased financial risks caused by the loss of health insurance [30] are also reasonable explanations for this phenomenon. This is a very difficult issue. On the one hand, avoiding hospitals leaves patients unable to receive necessary medical services. On the other hand, hospital managers encounter challenges in allocating resources, which may result in the less than optimal use of resources or a lack of resources. Previous studies identified the phenomenon of decline in outpatient numbers but did not provide targeted coping strategies. This cannot solve the problem because nobody knows when the COVID-19 pandemic will end. We must analyze and learn from existing experience, explore potential approaches, and plan for future emergency events. We believe that the reason previous studies fail to provide targeted coping strategies is that researchers have not conducted in-depth analyses of indicators that reflect the utilization of outpatient doctor resources. Therefore, we conducted a retrospective study of the change in outpatient numbers of 24 departments in the first 12 months (2020) of the COVID-19 pandemic, compared with the same period in 2019. Our goal was to understand the actual impact of the COVID-19 pandemic on different departments and provide suggestions to hospital managers regarding how to allocate outpatient resources more effectively during the pandemic. At the same time, we examined an indicator not mentioned in previous studies, which can reflect the utilization of outpatient doctor resources, the 2020 patient reservation rates of 24 departments. This provides more perspectives and possibilities for solving the resource allocation problem.

## 2. Materials and Methods

In this study, we collected 2019 and 2020 outpatient data for 24 departments at Peking University First Hospital, including the number of outpatients, number of reserved persons, and total number of reservation sources. We evaluated the impact of the COVID-19 pandemic on different departments by comparing outpatient numbers in 2019 and 2020. At the same time, we evaluated problems in the allocation of outpatient resources in different departments by calculating their patient reservation rates for different months in 2020. We used a matrix composed of outpatient change rates and patient reservation rates to provide targeted coping strategies for hospital managers to allocate outpatient doctor resources effectively under the influence of COVID-19.

### 2.1. Data Sources

Each department’s 2019 and 2020 outpatient data were retrospectively collected from the outpatient management information system of the Beijing Source Electronic Information Technology Company and sorted.

### 2.2. Definitions of Indicators

The number of outpatients refers to the number of patients who successfully reserved outpatient services and visited the hospital.

The patient reservation rate = (Number of persons who reserved outpatient services/ Total number of reservation sources) × 100%; the number of persons who reserved outpatient services is the number of successful reservations made by outpatients in each department through various reservation channels during the investigation period. Each department provided the number of reservation channels it used through all reservation sources during the investigation period.

### 2.3. Data Analysis 

A descriptive statistical analysis of the number of patients in 24 departments in 2019 and 2020 and change rates in patient numbers from 2019 and 2020 was undertaken using SPSS 24.0. At the same time, descriptive statistical analysis was carried out on the overall patient reservation rate and the monthly patient reservation rate in 24 departments.

Statisticians suggested that as this study includes data for all 24 departments of the hospital for two entire years (2019 and 2020), our data represent a whole, rather than a sample, of a few months. In this case, our data are not suitable for all types of hypothesis testing; therefore, we did not use specific statistical methods but did use descriptive analysis to compare and analyze the results. 

## 3. Results

### 3.1. Number and Change Rates of Patients in Various Departments, 2019 and 2020

The number of outpatients in all 24 departments combined decreased from 2,445,515 in 2019 to 1,629,619 in 2020, a decrease of 33.36%. In 10 departments, the number of outpatients fell by more than 33.36%, and in 12 departments, the number decreased by less than 33.36%; however, the number of outpatients in the tumor chemotherapy and radiotherapy departments increased in 2020 (Table 1).

### 3.2. Departmental Patient Reservation Rates 

**Figure 1:**
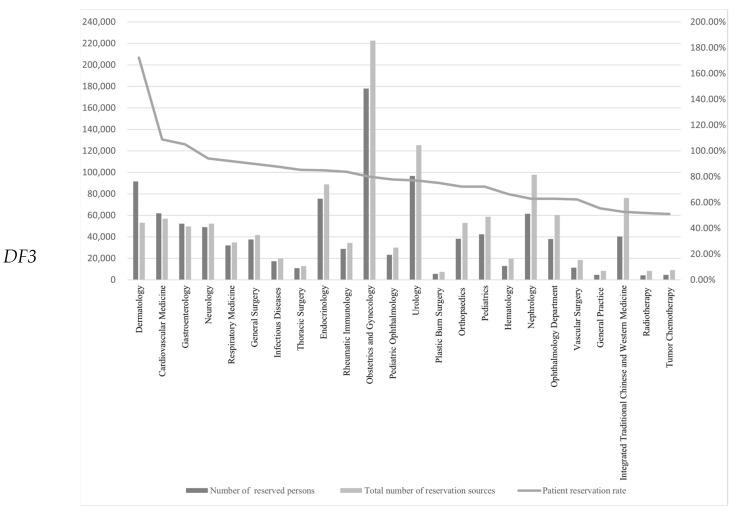
Patient reservation rates in each department.

The combined 2020 patient reservation rate in 24 departments was 82.22% of the 2019 reservation rate; in 14 departments, it was less than 82.22%. Four departments’ patient reservation rates were less than 60%: general medicine, integrated traditional Chinese and Western medicine, radiotherapy, and oncology chemotherapy. Ten departments (41.67%) had a 2020 patient reservation rate greater than 82.22% of the 2019 rate; in three departments (12.5%), dermatology and venereal diseases, cardiovascular medicine, and digestive medicine, the 2020 patient reservation rate exceeded 100% of the 2019 rate (Figure 1).

Note: In some departments, the utilization rate of outpatient reservations exceeded 100% because the number of reserved persons was greater than the total number of reservation sources actually provided by the department; doctors provide services for extra patients over time.

### 3.3. Monthly Departmental Reservation Rates

Table 2 show that the patient reservation rate for each department varied monthly. In 4 departments (16.67%), the monthly utilization rate of outpatient reservations did not exceed 82.22% in any month, and in 10 departments (41.67%), it did not exceed 82.22% in at least 10 months.

### 3.4. Outpatient Doctors Resource Allocation Strategy Matrix

**Figure 2:**
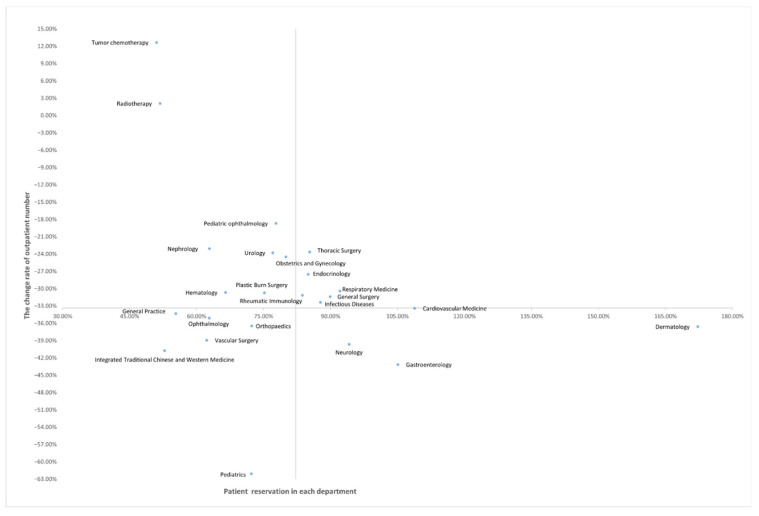
Outpatient doctor resource allocation strategy matrix.

Taking the patient reservation rate as the abscissa, the outpatient number change rate in 2020 compared with 2019 as the ordinate, and the coordinate value of the intersection point of the abscissa and ordinate (82.22%, −33.36%), the outpatient doctor resource allocation strategy matrix was obtained. In six departments, the decline in outpatient numbers was lower than average, and patient reservation rates were higher than average. In eight departments, the decline in outpatient numbers was lower than the average, and the patient reservation rates were higher than the average. In six departments, the decline in outpatient numbers was higher than average, and patient reservation rates were lower than average. In four departments, the decline in outpatient numbers was higher than average, as were patient reservation rates (Figure 2).

## 4. Discussion

We found that previous studies only focused on a specific department and rarely used key data regarding patient reservation rates. This is not conducive to the effective planning of outpatient doctor resources by hospital managers. As far as we know, our study is the first to include 24 different departments. Moreover, we not only focused on the change in outpatient numbers before and during the COVID-19 pandemic but also included patient reservation rates, which provides a strategy matrix that divides all departments into four categories, as shown in Figure 2. At the same time, we also analyze patient reservation rates in different months. This provides more perspectives and possibilities to solve the problem of how to allocate outpatient resources more efficiently. 

On the one hand, we found that compared with 2019, the annual outpatient number of 24 departments combined decreased by 33.36% in 2020. This result is consistent with many previous studies [9,10,11,12,13,14,15,16,17,18,19,20,21,22]. On the other hand, we have several additional findings specific to COVID-19’s impact on multiple hospital departments.

First, there is evidence that the initial impact of the COVID-19 pandemic on health systems was predominantly due to restriction policies rather than COVID-19 [23,24]. However, fear of COVID-19 prevents patients from attending hospitals during the pandemic [28]; given this, it is understandable that the overall number of hospital outpatients has greatly decreased. However, our study found a phenomenon that has not been reported elsewhere: the tumor chemotherapy and radiotherapy departments’ outpatient numbers increased. One possible explanation for this is that every person’s choice is a comprehensive result of balancing advantages and disadvantages [31], particularly with regard to personal health issues, and that people have become more cautious. Although the novel coronavirus pneumonia is a highly contagious and highly harmful infectious disease, patients in the oncology chemotherapy and radiotherapy departments have significant particularities compared with the general population; namely, they are suffering from various types of tumors. It is well known that for humans, tumors are one of the most significant medical challenges [32]. If many tumor patients do not receive effective and regular treatment in a timely manner, they may lose their lives in a short time; therefore, although there is a risk of COVID-19 infection, tumor patients are more willing to ‘expose themselves to danger’ than other patients. As no other study has reported similar results to date, we do not know whether this phenomenon exists in other countries. This is a useful reminder that the demand for medical care for patients with serious diseases, such as cancer, will not diminish in the face of serious infectious diseases, such as COVID-19; policymakers and hospital managers need to think about how to protect the interests of these patients in public health emergencies.

Second, although outpatient numbers for the other 22 departments decreased, differences in COVID-19’s impact on these departments are relatively large. Most departments with outpatient numbers that decreased less than the average were also the dominant departments in Peking University First Hospital, while departments whose outpatient numbers decreased more than the average were not well-known departments (except pediatrics and dermatology). Therefore, we want to emphasize that although managers of large general hospitals should be prepared for a decline in outpatient numbers in each department during the COVID-19 pandemic period, those departments with advantages and high visibility will be less affected. In contrast, for example, we believe that the numbers of emergency and severe cases in the dermatology department are relatively small, so patients are more likely to choose to delay treatments in this department when facing the risk of infection [33]. Similarly, because parents are particularly concerned about their children’s health, during the non-pandemic period, they were more likely to bring a child to the hospital when the child’s symptoms may not have required a doctor’s immediate attention [34]. However, it is reasonable to expect that, during the pandemic, parents wanting to avoid the risk of infection will only take a child to see a doctor when they think that the child’s symptoms require immediate medical intervention. This possibly explains the large decline in the number of pediatric patients from 2019 to 2020.

Third, when we combine data for patient reservation rates, we find a phenomenon worthy of attention. In the departments of dermatology and venereal diseases, cardiovascular medicine, and gastroenterology, outpatient numbers decreased more than the average for all the departments combined; however, patient reservation rates were higher than 100%. In contrast, outpatient numbers increased in the oncology chemotherapy and radiotherapy departments, but their patient reservation rates were the lowest. In the absence of data to guide decision-making, hospital managers cannot accurately determine the impact of the pandemic on different departments and thus cannot accurately allocate outpatient resources. As a result, resource supply shortages and resource supply exceeding demand occur simultaneously in different departments, which will have a significant impact on hospitals during a particularly difficult period, such as a pandemic. In addition, monthly patient reservation rates in some departments exhibited time aggregation. For example, pediatric monthly patient reservation rates were significantly higher in January; respiratory medicine monthly reservation rates were higher in February and from September to December; the plastic surgery monthly reservation rate for burns was highest in February; digestive medicine reservation rates were higher in January and February and from September to December; pediatric ophthalmology rates were higher in January and from July to September. This is partly because some diseases, such as respiratory diseases, are seasonal and more likely to occur in spring and winter [35]. External factors also help explain this phenomenon. For example, most people use hot water, stoves, and possibly fireworks more often during the Chinese Spring Festival (usually in February), which greatly increases the possibility of burns [36]. Similarly, many parents choose to treat their children’s various ophthalmic diseases when they have sufficient time during winter and summer vacations [37]. In addition, the pandemic’s outbreak phases and corresponding governmental prevention and control strategies may also have affected outpatient numbers in different months. Many factors need to be considered to more accurately analyze outpatient numbers in different departments during the pandemic.

Therefore, according to the decision matrix shown in Figure 2 and the patient reservation rates in different months, we make the following recommendations for the allocation of outpatient resources during the pandemic.

First, rather than rely on human experience, it is better to have and use objective indicators [38]. Hospital managers should transition from ‘experience-based’ to ‘data-based’ decisions to facilitate comprehensive, objective, and quantitative management of outpatient doctor allocation; this will help achieve a better balance between resource supply and demand. In departments where the decline in outpatient numbers was lower than average, and patient reservation rates were higher than average (as shown in Figure 2), we suggest that existing outpatient resources can be maintained. In departments where the decline in outpatient numbers and patient reservation rates were both lower than the average, we suggest that the allocation of outpatient doctors can be appropriately reduced. In departments where both the declines in outpatient numbers and patient reservation rates were higher than average, we suggest providing patients with advisory services through Internet medical services as well as considering improving the resource allocation of offline doctors to a small extent. Finally, in departments where the decline in outpatient numbers was higher than average, and patient reservation rates were lower than average, we propose significantly redeploying the allocation of doctor resources to other more important tasks. Similarly, as monthly patient reservation rates vary both in and across different departments, we suggest that departmental doctor attendance should be flexible; that is, more doctors should be allocated in months with a high patient utilization rate, while fewer doctors should be allocated in other months [36]. 

Second, it should be emphasized to hospital managers that the principles of supply and demand will not remain unchanged as the pandemic changes very rapidly. While some researchers claim that mathematical models for optimal SARS-CoV-2 eradication strategies have been developed, such strategies depend on many prerequisites, such as systematic planning, effective hospital isolation, SARS-CoV-2 vaccination, and other measures, including urban closures and leave policies [39], making it difficult to accurately predict pandemic development trends. Therefore, COVID-19’s impact on different hospital departments may also fluctuate. We suggest that the management of outpatient doctors requires sufficient flexibility, as well as paying attention to departmental changes in patient reservation rates over time to enable dynamic adjustments in response.

Finally, we emphasize that doctors have more social responsibilities and public expectations than many other professions. While the vast majority of doctors have made many sacrifices because of these expectations, it cannot be ignored that doctors have emotions, personal working habits and preferences, and a variety of needs, as identified in Hirotaka’s study of the impact of post-surgery patient mortality on the surgeon’s day [40]. Especially in such an unusual period as the pandemic, solutions to many problems depend not only on resources, management strategies, action, and so on; humanistic care must also be taken into account. Studies have shown that medical staff on the anti-pandemic front line suffer multiple pressures from unknown diseases, high-intensity work, external public opinion, patients, and their families [41,42]. For example, a study of American obstetrics and gynecology doctors confirmed that during the COVID-19 pandemic, medical staff experienced sympathetic fatigue and common trauma due to their proximity to the pain experienced by patients [43]. Studies from China, Italy, and Canada reported that medical staff experienced severe depression, anxiety, insomnia, and pain in the treatment of COVID-19 patients [44,45,46]. It is important to realize that doctors are very often deprived of their basic rights and protections in the workplace; their exploitation is rationalized based on the belief that medicine needs to sacrifice itself [47]. Therefore, hospital and department managers need to have a spirit of humanistic care in the management of human resource allocation, giving due consideration to doctors’ personal needs, working habits, and special circumstances, rather than relying solely on cold data and models to force doctors’ attendance at a fixed time.

Overall, our findings are valuable to hospital managers, who need to allocate outpatient doctors effectively during the COVID-19 pandemic. First, although overall hospital outpatient numbers declined because of the impact of COVID-19, higher-profile departments were less affected by this than other departments, and cancer-related departments had increased outpatient numbers. Second, we examined an indicator not mentioned in previous studies, patient reservation rates. Compared with changes in outpatient numbers, patient reservation rates are the real indicator of the utilization of outpatient doctor resources. Therefore, we suggest that hospital managers pay attention to this indicator at the departmental level when allocating resources. Finally, we propose a resource allocation strategy that can be used by hospital managers, which is precise, dynamic, and humanized.

This study has some shortcomings. First, our data were from one hospital; therefore, some conclusions may not be widely applicable to all hospitals. However, we believe that hospital managers around the world can use our precise, dynamic, humanized strategy as a reference. Second, valuable information may be obtained by analyzing the gender, age, and disease type of patients in different departments. This will be a massive and lengthy task best undertaken separately for specific departments rather than at the same time for 24 departments. Third, interviewing patients may provide a better understanding of why people stopped visiting the hospital, and specific departments, during the pandemic. However, face-to-face interviews are not possible given the current pandemic; this will be a worthwhile and interesting research project in future. Finally, we expect to use the matrix obtained in this study for future management practice and to optimize it continuously. For example, we can use this matrix to optimize the allocation of outpatient doctor resources in Peking University First Hospital, as well as other hospitals willing to participate in the study. After six months or one year, we could evaluate the rationality and effectiveness of the matrix by assessing whether the index of patient reservation rates has been optimized.

## 5. Conclusions

The COVID-19 pandemic continues to have a substantial impact on global health institutions, bringing unprecedented challenges to hospital managers allocating medical resources. However, in large general hospitals, each department is affected differently. Hospital managers can ensure the effective use of resources by dynamically and meticulously managing departmental outpatient doctor resources in response to changes in outpatient numbers and patient reservation rates. At the same time, it is necessary to show humanistic care to doctors when allocating human resources.

## Figures and Tables

**Table 1:** Number of outpatients in each department in 2019 and 2020.

Departments	2019	2020	Change Rates in 2020 Compared with 2019
Pediatrics	190,937	72,425	−62.07%
Radiotherapy	12,734	13,000	2.09%
Rheumatic Immunology	53,410	36,777	−31.14%
Obstetrics and Gynecology	393,131	296,837	−24.49%
Infectious Diseases	34,026	23,013	−32.37%
Orthopaedics	103,955	66,045	−36.47%
Respiratory Medicine	65,602	45,641	−30.43%
Vascular Surgery	27,674	16,898	−38.94%
Urology	191,259	145,749	−23.79%
Endocrinology	139,177	100,863	−27.53%
Dermatology	239,615	151,967	−36.58%
General Surgery	139,468	95,704	−31.38%
General Practice	37,469	24,612	−34.31%
Neurology	130,920	79,015	−39.65%
Nephrology	137,496	105,757	−23.08%
Gastroenterology	117,214	66,635	−43.15%
Pediatric Ophthalmology	36,794	29,918	−18.69%
Cardiovascular Medicine	131,738	87,718	−33.41%
Thoracic Surgery	20,812	15,892	−23.64%
Hematology	24,064	16,685	−30.66%
Ophthalmology	83,823	54,403	−35.10%
Plastic Burn Surgery	13,825	9578	−30.72%
Integrated Traditional Chinese and Western Medicine	113,640	67,354	−40.73%
Tumor Chemotherapy	6332	7133	12.65%
Total	2,445,515	1,629,619	−33.36%

**Table 2:** Monthly departmental patient reservation rates.

Departments	Jan	Feb	Mar	Apr	May	Jun	Jul	Aug	Sept	Oct	Nov	Dec
Pediatrics	112.86%	55.65%	69.66%	59.41%	69.91%	56.74%	65.34%	77.32%	76.09%	74.15%	71.32%	68.01%
Radiotherapy	24.15%	41.29%	62.86%	54.26%	56.17%	50.88%	55.58%	57.55%	59.12%	59.51%	52.40%	52.39%
Rheumatic Immunology	61.83%	89.34%	82.15%	82.43%	73.67%	63.59%	76.40%	93.83%	96.20%	101.92%	93.32%	87.30%
Obstetrics and Gynecology	73.04%	51.18%	70.29%	69.17%	79.57%	73.09%	74.61%	79.96%	82.72%	87.25%	93.83%	105.00%
Infectious Diseases	31.47%	70.65%	86.42%	87.32%	142.57%	128.00%	145.74%	210.09%	237.87%	98.85%	66.62%	73.18%
Orthopedics	57.46%	65.22%	86.10%	65.01%	80.75%	63.68%	70.54%	71.48%	76.81%	84.71%	73.25%	71.92%
Respiratory Medicine	78.76%	110.46%	95.51%	81.99%	78.08%	76.07%	78.96%	85.30%	106.41%	103.76%	108.81%	128.11%
Vascular Surgery	47.08%	74.27%	60.75%	56.13%	67.95%	64.66%	59.36%	70.68%	73.55%	73.70%	57.98%	55.05%
Urology	75.38%	60.60%	89.50%	66.94%	75.27%	70.68%	72.13%	80.15%	77.93%	83.94%	82.91%	81.49%
Endocrinology	102.62%	94.41%	70.91%	71.49%	82.30%	71.50%	78.04%	89.38%	91.46%	91.80%	92.01%	89.93%
Dermatology	105.96%	135.46%	103.14%	108.27%	175.77%	140.34%	154.83%	192.37%	226.89%	235.57%	203.48%	192.83%
General Surgery	75.03%	119.72%	80.24%	70.94%	91.89%	78.81%	83.90%	93.43%	105.88%	99.69%	94.72%	91.81%
General Practice	50.28%	60.00%	50.00%	33.76%	39.24%	49.72%	50.13%	64.63%	45.51%	53.11%	68.78%	88.42%
Neurology	87.03%	90.42%	80.85%	74.02%	81.80%	87.16%	97.04%	100.13%	106.31%	106.35%	105.58%	97.90%
Nephrology	64.20%	47.74%	54.26%	53.51%	60.94%	57.76%	54.70%	63.59%	67.13%	74.39%	68.73%	67.94%
Gastroenterology	114.96%	144.37%	83.25%	82.43%	90.37%	88.21%	93.87%	94.27%	102.13%	111.15%	126.34%	133.00%
Pediatric Ophthalmology	92.45%	20.00%	61.45%	70.07%	87.38%	60.38%	82.71%	94.71%	89.87%	78.61%	70.96%	65.85%
Cardiovascular Medicine	112.20%	261.43%	121.27%	132.22%	116.87%	88.90%	97.97%	103.36%	98.16%	113.05%	107.45%	105.13%
Thoracic Surgery	53.16%	150.67%	85.84%	84.08%	80.75%	78.56%	91.53%	97.40%	92.38%	87.27%	95.93%	94.00%
Hematology	65.56%	137.50%	82.29%	71.56%	84.23%	52.05%	50.00%	67.04%	66.65%	70.29%	66.31%	69.03%
Ophthalmology	39.42%	53.17%	79.61%	79.83%	62.02%	56.18%	64.44%	63.14%	74.39%	64.65%	72.04%	60.80%
Plastic Burn	63.16%	102.22%	88.09%	81.96%	70.61%	73.72%	70.39%	77.30%	78.06%	76.50%	78.17%	75.80%
Integrated Traditional Chinese and Western Medicine	33.97%	110.08%	63.24%	46.87%	53.22%	53.73%	58.38%	53.44%	56.36%	56.33%	53.01%	55.66%
Tumor Chemotherapy	11.27%	84.23%	40.58%	39.34%	56.72%	60.30%	64.85%	66.41%	74.29%	77.36%	57.75%	51.11%

Note: In some departments, the monthly patient reservation rate exceeded 100% because the number of registered persons was greater than the total number of reservation sources actually provided by the department; doctors provide outpatient services for extra patients through overtime.

## Data Availability

Not applicable.

## References

[1] COVID-19 Situation Updates Worldwide, as of 15 October 2020. European Centre for Disease Prevention and Control. https://www.ecdc.europa.eu/en/geographical-distribution-2019-ncov-cases.

[2] Zhu N., Zhang D., Wang W., Li X., Yang B., Song J., Zhao X., Huang B., Shi W., Lu R. (2020). A Novel Coronavirus from Patients with Pneumonia in China, 2019. N. Engl. J. Med..

[3] How to Protect Yourself & Others. Centers for Disease Control and Prevention. https://www.cdc.gov/coronavirus/2019-ncov/prevent-getting-sick/prevention.html.

[4] Kishore S., Hayden M. (2020). Community Health Centers and COVID-19 -Time for Congress to Act. N. Engl. J. Med..

[5] Khullar D., Bond A.M., Schpero W.L. (2020). COVID-19 and the Financial Health of US Hospitals. JAMA.

[6] Sohrabi C., Alsafi Z., O’Neill N. (2020). World Health Organization declares global emergency: A review of the 2019 novel coronavirus (COVID-19). Int. J. Surg..

[7] Viale G., Licata L., Sica L., Zambelli S., Zucchinelli P., Rognone A., Aldrighetti D., Di Micco R., Zuber V., Pasetti M. (2020). Personalized risk-benefit ratio adaptation of breast cancer care at the epicenter of COVID-19 outbreak. Oncologist.

[8] Cigăran R.-G., Botezatu R., Mînecan E.-M., Gică C., Panaitescu A.M., Peltecu G., Gică N. (2021). The Psychological Impact of the COVID-19 Pandemic on Pregnant Women. Healthcare.

[9] Lee H.-H., Lin S.-H. (2020). Effects of COVID-19 Prevention Measures on Other Common Infections, Taiwan. Emerg. Infect. Dis..

[10] Mehrotra A. What Impact Has COVID-19 Had on Outpatient Visits?. Commonwealth Fund..

[11] Thornton J. (2020). COVID-19: A&E visits in England fall by 25% in week after lockdown. BMJ.

[12] Murray R., Edwards N. Delivering Core NHS and Care Services during the COVID-19 Pandemic and beyond. The King’s Fund. https://www.kingsfund.org.uk/publications/letter-to-health-and-social-care-select-committee-COVID-19.

[13] Shih C.L., Huang P.J., Huang H.T., Chen C.H., Lee T.C., Hsu C.H. (2021). Impact of the COVID-19 pandemic and its related psychological effect on orthopedic surgeries conducted in different types of hospitals in Taiwan. J. Orthop. Surg..

[14] Dai Y.X., Ma S.H., Tai Y.H., Chen C.C., Chen T.J., Chang Y.T. (2020). Impact of the COVID-19 pandemic on dermatology clinic visits: Experience from a tertiary medical center in Taiwan. Dermatol. Sin..

[15] Savastano A., Ripa M., Savastano M.C., Kilian R., Marchini G., Rizzo S. (2021). Impact of the COVID-19 pandemic on ophthalmologic outpatient care: Experience from an Italian Tertiary Medical Center. Ann. Med..

[16] Xie T., Wang D., Wang X., Yang Q., Sun H., Liu R., Li M. (2021). Impact of COVID-19 pandemic on outpatient reservations of rheumatic patients in anon-outbreak area of China. Wien. Klin. Wochenschr..

[17] Vogel M., Beger C., Gausche R., Jurkutat A., Pfaeffle R., Körner A., Meigen C., Poulain T., Kiess W. (2021). COVID-19 Pandemic and Families Utilization of Well Child Clinics and Pediatric Practice Attendance in Germany. BMC Res. Notes.

[18] Moharrami M., Bohlouli B., Amin M. (2022). Frequency and Pattern of Outpatient Dental Visits during the COVID-19 Pandemic at Hospital and Community Clinics. J. Am. Dent. Assoc..

[19] Daniel P., Johannes K., Lukas G., Demetz M., Hartmann S., Thomé C. (2021). Effect of the COVID-19 Pandemic on Patient Presentation and Perception to a Neurosurgical Outpatient Clinic. World Neurosurg..

[20] Jennifer N., Pratima A., David O., Melissa G., Jacob B., Jacquelyn P., Lauren F., Jessica R. (2021). Effects of COVID-19 on telemedicine practice patterns in outpatient otolaryngology. Am. J. Otolaryngol..

[21] Rosenberg S., Mendoza J., Tabatabaei-Jafari H., Salvador-Carulla L., Pandemic-Mental Health International Network (Pan-MHIN) (2020). International experiences of the active period of COVID-19—Mental health care. Health Policy Technol..

[22] Townsend J.W., Ten Hoope-Bender P., Sheffield J. (2020). In the response to COVID-19, we can’t forget health system commitments to contraception and family planning. Int. J. Gynaecol. Obstetr..

[23] Zhang J., Litvinova M., Liang Y., Wang Y., Wang W., Zhao S., Wu Q., Merler S., Viboud C., Vespignani A. (2020). Changes in contact patterns shape the dynamics of the COVID-19 outbreak in China. Science.

[24] Civil Protection Department, Government of Italy Chronology of Main Steps and Legal Acts Taken by the Italian Government for the Containment of the COVID-19 Epidemiological Emergency. http://www.protezionecivile.gov.it/documents/20182/1227694/Summary+of+measures+taken+against+the+spread+of+C-19/c16459ad-4e52-4e90-90f3-c6a2b30c17eb..

[25] Hu X., Liu S., Wang B., Xiong H., Wang P. (2020). Management practices of emergency departments in general hospitals based on blockage of chain of infection during a COVID-19 epidemic. Intern. Emerg. Med..

[26] Paterlini M. (2020). On the front lines of coronavirus: The Italian response to COVID-19. BMJ.

[27] Webster P. (2020). Virtual health care in the era of COVID-19. Lancet.

[28] Kartal S.P., Çelik G., Sendur N., Aytekin S., Serdaroğlu S., Doğan B., Yazıcı A.C., Çiçek D., Borlu M., Kaçar N.G. (2020). Multicenter study evaluating the impact of COVID-19 outbreak on dermatology outpatients in Turkey. Derm. Ther..

[29] Garcia S., Albaghdadi M.S., Meraj P.M., Schmidt C., Garberich R., Jaffer F.A., Dixon S., Rade J.J., Tannenbaum M., Chambers J. (2020). Reduction in ST-Segment Elevation Cardiac Catheterization Laboratory Activations in the United States During COVID-19 Pandemic. . J. Am. Coll. Cardiol..

[30] Kocher K.E., Macy M.L. (2020). Emergency Department Patients in the Early Months of the Coronavirus Disease 2019 (COVID-19) Pandemic-What Have We Learned?. JAMA Health Forum..

[31] Bravo A.J., Pearson M.R., Stevens L.E., Henson J.M. (2018). Weighing the Pros and Cons of Using Alcohol Protective Behavioral Strategies: A Qualitative Examination among College Students. Subst. Use Misuse.

[32] Are C., Rajaram S., Are M., Raj H., Anderson B.O., Swamy R.C., Vijayakumar M., Song T., Pandey M., Edney J.A. (2013). A review of global cancer burden: Trends, challenges, strategies, and a role for surgeons. J. Surg. Oncol..

[33] Alfieri A., Yogianti F. (2021). Impact of pandemic COVID-19 on dermatology and venereology outpatient clinic in a tertiary referral hospital in Yogyakarta, Indonesia. Derm. Rep..

[34] Voigt R.G., Johnson S.K., Hashikawa A.H., Mellon M.W., Campeau L.J., Williams A.R., Yawn B.P., Juhn Y.J. (2008). Why Parents Seek Medical Evaluations for Their Children With Mild Acute Illnesses. Clin. Pediatr..

[35] Jiang Y., Chen J., Wu C., Lin X., Zhou Q., Ji S., Yang S., Zhang X., Liu B. (2020). Temporal cross-correlations between air pollutants and outpatient visits for respiratory and circulatory system diseases in Fuzhou, China. BMC Public Health.

[36] Wu H., Xiao Q. (2022). Analysis of seasonal index variation of admissions in various professional wards of a hospital. China Health Stat..

[37] Gan X. (2020). The practice of applying quality control circle to shorten the visiting time of adolescent optometry patients in holiday clinics. Integr. Chin. West. Med. Nurs..

[38] Jiang M., Ma Y., Guo S., Jin L., Lv L., Han L., An N. (2021). Using Machine Learning Technologies in Pressure Injury Management: Systematic Review. JMIR Med. Inform..

[39] Jiang S., Li Q., Li C., Liu S., He X., Wang T., Li H., Christopher C., Zhang X., Xu J. (2020). Mathematical models for devising the optimal SARS-CoV-2 strategy for eradication in China, South Korea, and Italy. J. Transl. Med..

[40] Kato H., Jena A.B., Tsugawa Y. (2020). Patient mortality after surgery on the surgeon’s birthday: Observational study. BMJ.

[41] World Health Organization (2020). Coronavirus Disease (COVID-19) Outbreak: Rights, Roles and Responsibilities of Health Workers, Including Key Considerations for Occupational Safety and Health..

[42] Shanafelt T., Ripp J., Trockel M. (2020). Understanding and addressing sources of anxiety among health care professionals during the COVID-19 Pandemic. JAMA.

[43] Werner E.A., Aloisio C.E., Butler A.D., D’Antonio K.M., Kenny J.M., Mitchell A., Ona S., Monk C. (2020). Addressing mental health in patients and providers during the COVID-19 pandemic. Semin. Perinatol..

[44] Lai J., Ma S., Wang Y., Cai Z., Hu J., Wei N., Wu J., Du H., Chen T., Li R. (2020). Factors associated with mental health outcomes among health care workers exposed to coronavirus disease 2019. JAMA Netw. Open.

[45] Rossi R., Socci V., Pacitti F., Di Lorenzo G., Di Marco A., Siracusano A., Rossi A. (2020). Mental health outcomes among front and second line health workers associated with the COVID-19 pandemic in Italy. JAMA Netw Open..

[46] Potloc Potloc Study: Canadian Health Workers Share Their Insights from the Front Lines of the COVID-19 Pandemic. https://www.potloc.com/blog/en/potloc-study-canadian-health-workers-insights-front-lines-covid-19-pandemic.

[47] Arnold-Forster A., Moses J.D., Schotland S.V. (2022). Obstacles to Physicians’ Emotional Health—Lessons from History. N. Engl. J. Med..

